# Spatially Explicit Modeling of Schistosomiasis Risk in Eastern China Based on a Synthesis of Epidemiological, Environmental and Intermediate Host Genetic Data

**DOI:** 10.1371/journal.pntd.0002327

**Published:** 2013-07-25

**Authors:** Matthias Schrader, Torsten Hauffe, Zhijie Zhang, George M. Davis, Fred Jopp, Justin V. Remais, Thomas Wilke

**Affiliations:** 1 Department of Animal Ecology and Systematics, Justus Liebig University Giessen, Giessen, Germany; 2 Department of Epidemiology and Biostatistics, School of Public Health, Fudan University, Shanghai, People's Republic of China; 3 Department of Microbiology and Tropical Medicine, George Washington University Medical Center, Washington, District of Columbia, United States of America; 4 Department of Environmental Health, Rollins School of Public Health, Emory University, Atlanta, Georgia, United States of America; University of Queensland, Australia

## Abstract

Schistosomiasis japonica is a major parasitic disease threatening millions of people in China. Though overall prevalence was greatly reduced during the second half of the past century, continued persistence in some areas and cases of re-emergence in others remain major concerns. As many regions in China are approaching disease elimination, obtaining quantitative data on *Schistosoma japonicum* parasites is increasingly difficult. This study examines the distribution of schistosomiasis in eastern China, taking advantage of the fact that the single intermediate host serves as a major transmission bottleneck. Epidemiological, population-genetic and high-resolution ecological data are combined to construct a predictive model capable of estimating the probability that schistosomiasis occurs in a target area (“spatially explicit schistosomiasis risk”). Results show that intermediate host genetic parameters are correlated with the distribution of endemic disease areas, and that five explanatory variables—altitude, minimum temperature, annual precipitation, genetic distance, and haplotype diversity—discriminate between endemic and non-endemic zones. Model predictions are correlated with human infection rates observed at the county level. Visualization of the model indicates that the highest risks of disease occur in the Dongting and Poyang lake regions, as expected, as well as in some floodplain areas of the Yangtze River. High risk areas are interconnected, suggesting the complex hydrological interplay of Dongting and Poyang lakes with the Yangtze River may be important for maintaining schistosomiasis in eastern China. Results demonstrate the value of genetic parameters for risk modeling, and particularly for reducing model prediction error. The findings have important consequences both for understanding the determinants of the current distribution of *S. japonicum* infections, and for designing future schistosomiasis surveillance and control strategies. The results also highlight how genetic information on taxa that constitute bottlenecks to disease transmission can be of value for risk modeling.

## Author Summary

Schistosomiasis is considered the second most devastating parasitic disease after malaria. In China, it is transmitted to humans, cattle and other vertebrate hosts by a single intermediate snail host. It has long been suggested that the close co-evolutionary relationship between parasite and intermediate host makes the snail a major transmission bottleneck in the disease life cycle. Here, we use a novel approach to model the disease distribution in eastern China based on a combination of epidemiological, ecological, and genetic information. We found four major high risk areas for schistosomiasis occurrence in the large lakes and flood plain regions of the Yangtze River. These regions are interconnected, suggesting that the disease may be maintained in eastern China in part through the annual flooding of the Yangtze River, which drives snail transport and admixture of genotypes. The novel approach undertaken yielded improved prediction of schistosomiasis disease distribution in eastern China. Thus, it may also be of value for the predictive modeling of other host- or vector-borne diseases.

## Introduction

Schistosomiasis japonica is a major parasitic disease threatening 50–65 million people living in subtropical areas of China [1]. Though overall prevalence and intensity of infection were reduced by more than 90% during the second half of the past century [2], [3], the possibility of continued reduction of schistosomiasis to achieve rapid elimination has recently been questioned [4]. Highly variable rates of reduction across counties, continued persistence in some areas, and cases of re-emergence in others, remain major concerns [3], [5], [6]. The conditions that characterize the current, critical stage of disease elimination in China call for new strategies in disease surveillance and control [7]. The current control target aimed at reducing human and bovine infection rates in all endemic counties to less than 1% by 2015 [8]–[11] largely focuses on morbidity control. This strategy could benefit from the inclusion of evolutionary and ecological perspectives, particularly as concerns key epidemiological and surveillance concepts.

For instance, a basic epidemiological concept used in China's schistosomiasis surveillance and control strategy is ‘endemic area’. It refers to a region where a particular disease is prevalent [12] based on standardized parameters, mainly rates of infection in residents and/or cattle [13]. It does not explicitly consider evolutionary aspects such as the relative spatial isolation of populations transmitting the disease [7], [14]. Such an evolutionary (i.e., population-based) approach, however, could help shift capabilities from simply analyzing the patterns of disease transmission to understanding the actual processes responsible for generating these patterns.

Another instance where an evolutionary perspective could be useful relates to the fact that China's current control strategy primarily targets only two main hosts, humans and cattle [15], even though more than 40 mammalian species are known to serve as definitive host [7]. Therefore, it is reasonable to assume that cases of cryptic persistence of schistosomiasis are common, calling for approaches focusing on the snail host as major transmission bottleneck in the schistosome life cycle (“no snails, no disease”) [16].

Unlike other snail-schistosome models, *Schistosoma japonicum* is carried by a single species of snail with a peculiar amphibious life style, and there are no known cases of snail host-switching and/or host-addition in China [16]. This makes *Oncomelania hupensis* a crucial target for disease control. Empirical evidence even suggests a close link between snail genetic characters and rates of infection in snails [16]–[19], a possible consequence of the effects of positive frequency dependent selection, e.g., genetic warfare between host and parasite leading to sustained oscillations in genotype frequencies [20]–[22].

Given the large number of definitive hosts for *S. japonicum*, and the fact that quantitative parasite data are increasingly difficult to obtain due to low rates of natural infections [7], the intermediate snail host thus becomes of particular interest. Here, we take a fresh look at schistosomiasis distribution in eastern China from the evolutionary and ecological viewpoint of a single intermediate host system. For the first time, epidemiological with spatially explicit population-genetic and high-resolution ecological data are combined to develop a predictive model capable of estimating the probability that schistosomiasis occurs in a target area (here termed ‘spatially explicit schistosomiasis risk’—SESR). The study pursues four specific goals:

- We test whether genetic intermediate host characters (i.e., intrinsic evolutionary properties of populations), in principle, are reflected by previously defined endemic areas.
- We use a candidate set of topographical, ecological and genetic variables together with maximum entropy modeling to identify those explanatory characters that significantly discriminate between endemic/non-endemic areas.
- Based on the parameters with the highest discriminatory power, we then develop a SESR model, converting categorical infection data for administrative units into spatially explicit, high-resolution, and quantitative reaction data.
- Finally we compare the model data with actual human infection rates to evaluate our SESR model and to assess the significance of intermediate host traits for future epidemiological modeling of schistosomiasis.

## Materials and Methods

### Specimens Studied

**Figure 1:**
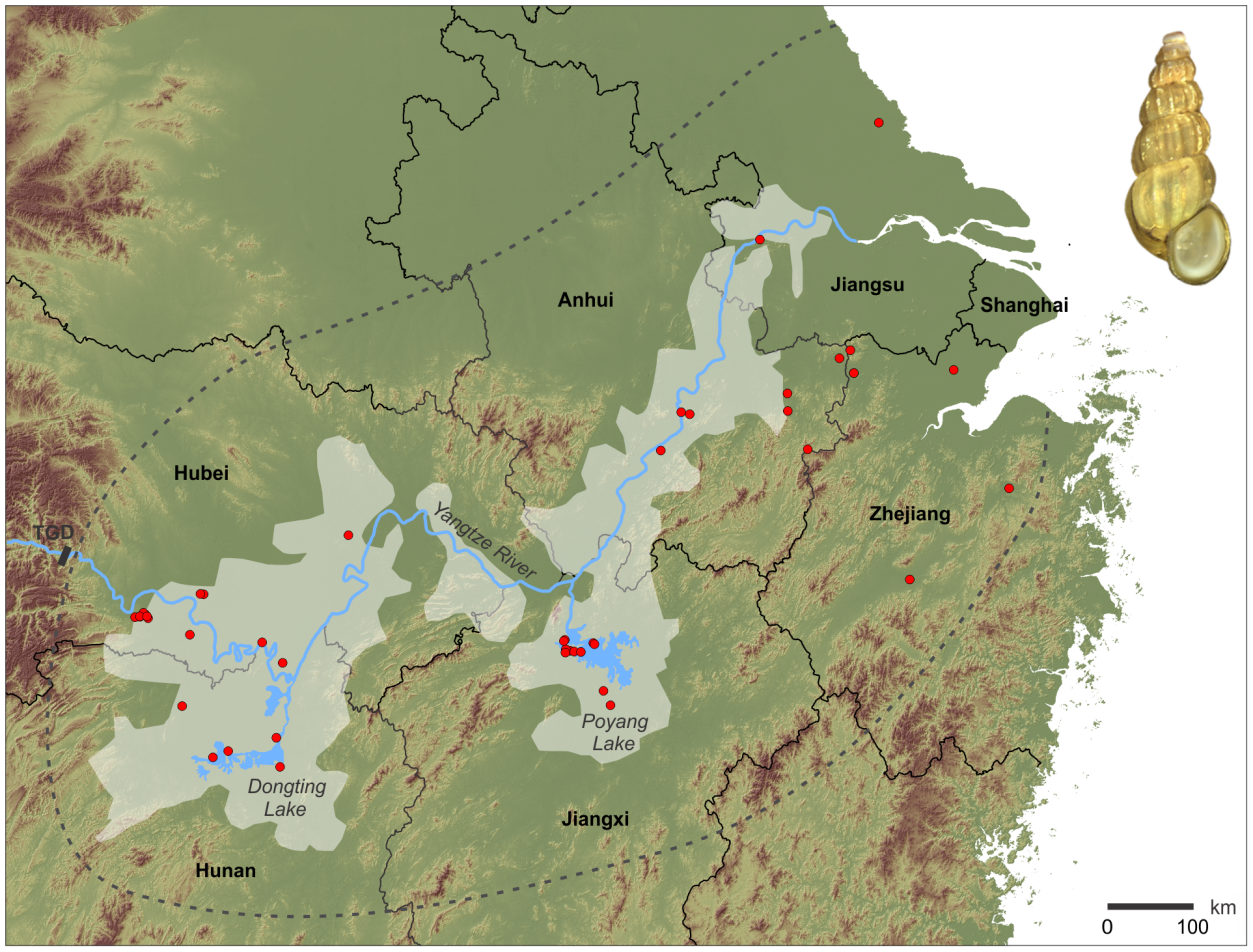
Schistosomiasis study area in eastern China. The map shows the localities of the intermediate snail host *Oncomelania h. hupensis* sampled (red dots), the assumed maximum distribution area of this subspecies in the lower Yangtze River basin (dashed gray line), and previously delineated endemic areas [5] (highlighted areas). The distribution area is based on our own sampling data and literature records [58], [74], [80], [81], restricted by a reasonable vertical distribution of 0 to 200 m a.s.l. [2]. For detailed locality information see Supporting Table S1. TGD = Three Gorges Dam.

Today, the human blood fluke *S. japonicum* (Katsurada, 1904) is transmitted in the Yangtze River area by two snail subspecies, *Oncomelania hupensis hupensis* Gredler, 1881 and *O. h. robertsoni* Bartsch, 1946. The two taxa (and therefore the disease as well) have disjunct ranges; the eastern subspecies *O. h. hupensis* occurs in the lowlands of the Yangtze River below the Three Gorges (Fig. 1); the western subspecies *O. h. robertsoni* in mountainous regions of Sichuan and Yunnan provinces.

This study includes 530 specimens of the eastern subspecies *O. h. hupensis* from 45 sites (‘populations’) in six Chinese provinces. Specimens were largely collected between 1996 and 2005 (plus three additional populations in 1984) in and around the lower Yangtze River basin. Thirty one of these sites are located within and fourteen outside of previously proposed endemic areas (Fig. 1; Supporting Table S1).

All specimens were obtained before the completion of the Three Gorges Dam (TGD) project in 2007. It is assumed that dam-associated changes in water regime and sedimentation rates of the lower Yangtze River will have significant effects on the distribution patterns of the intermediate host and thus disease distribution [10], [16], [23]. As these demographic processes are likely reflected by snail population structures [16], the data presented here may also serve as valuable baseline for future studies of pre- vs. post-dam effects on schistosomiasis in China.

### Molecular Data

#### DNA extraction, amplification and sequencing

Genomic DNA was extracted from individual snail specimens utilizing a CTAB protocol [24]. Digital images of selected specimens were taken prior to consumptive DNA isolation and deposited at the University of Giessen Systematics and Biodiversity collection (UGSB). We amplified a fragment of the mitochondrial cytochrome *c* oxidase subunit I (COI) gene with a target length of 658 base pairs (excluding primer sequence). Forward and reverse primers for PCR amplification and DNA sequencing were LCO1490 [25] and COR722b [26]; the latter is a modification of primer HCO2198 [26]. Bidirectional DNA sequencing according to the ‘Sanger’ chain-termination method [27] was performed either on a Long Read IR2 4200 sequencer (LI-COR, Lincoln, NE, USA) or an ABI3730XL sequencer (Life Technologies Corporation, Carlsbad, CA, USA). The protein-coding COI sequences, which are free of insertions and deletions in the family Pomatiopsidae [28], were aligned in BioEdit 7.1.3.0 [29]. As the first base pairs behind the 3′ end of each primer were difficult to read, we trimmed these regions, leaving a 638 bp-long overlapping fragment. All 440 newly generated sequences were deposited in GenBank. Additional 90 sequences were taken from GenBank, resulting in a total dataset of 530 sequences (Supporting Table S1).

### Correlation Analysis of Previously Defined Endemic Areas and Intermediate Host Genetics

In order to test whether genetic snail parameters, in principle, can be explained by the distribution of previously defined endemic areas (see Goal 1), we used two independent approaches. With an Analysis of Molecular Variance (Amova) [30], it was tested whether there is a significant partitioning of variance of genetic characters from populations within vs. populations outside of endemic areas. This analysis is not fully spatially-explicit as the overall geographical distribution of snail populations is not considered. Alternatively, we used Multivariate Regression Trees (MRT) [31] to test whether genetic characters can be predicted using endemic areas. This analysis differs from the Amova by being spatially-explicit (see below).

As our snail populations were collected over a period of approximately 20 years (see Supporting Table S1), we conducted two Amova tests (A, B). Amova A served as a pre-analysis to exclude the possibility of a sampling bias by testing for the presence of a significant partitioning of variance of genetic characters from populations being collected during different time periods. Amova B was then used to test for a partitioning of variance between populations from endemic vs. non-endemic areas. For the former analysis, our grouping variables were sampling periods (1980s, 1990s, and 2000s) according to Supporting Table S1. For the latter one, populations were grouped according to their assignment to endemic vs. non-endemic areas based on the endemic area distribution as previously suggested [5] (also see Fig. 1 and Supporting Table S1). Then, a distance matrix of pairwise nucleotide differences was calculated in Arlequin 3.5.1.2. [32] and the significance of the Φ statistic (α≤0.05) tested by generating a null-distribution based on 10,000 permutations of the original dataset.

The MRT approach used the principle coordinates obtained by multidimensional scaling of pairwise differences as response variables. The variables were standardized by dividing by the respective maximum, and endemic state and geographical coordinates served as explanatory variables (latitude and longitude information was converted into northing and easting according to the Asia North Equidistant Conic projection for obtaining equidistant values). As previously suggested [33], the first split of the tree can be forced for hypothesis testing. Accordingly, we pre-defined this split to discriminate between endemic and non-endemic areas. Hierarchical nesting for MRT was then done with the MVPARTwrap 0.1.8 package [33] for R 2.15 statistical environment [34]. The overall best tree was selected by running 1000 10-fold cross validations.

### SESR Modeling

#### Candidate snail traits

For identifying the genetic parameters that significantly reflect the spatial distribution of endemic areas, four candidate population indices were calculated from the COI dataset. They comprised within-site (‘diversity’) and between-site (‘divergence’) parameters. The first set of parameters consisted of nucleotide diversity π (average number of nucleotide differences per site within populations based on equation 10.5 in Nei [35]) and Tajima-Nei-distance D_TN_ (average number of nucleotide differences per site between populations, corrected for unequal rates of substitution [36]), calculated in the R statistical environment. The second set of indices utilized haplotype information, which was previously suggested to be correlated with infection rates in snails [2], [16], [19] (also see section “Predictive variables of SESR” in the Discussion). We calculated both haplotype diversity (H_D_) and haplotype divergence (H_MH_) in order to assess within and between population differentiations, respectively. H_D_ was estimated in DnaSP v5 based on equation 8.4 in Nei [35]. For calculating H_MH_, we treated haplotypes as species [37] and estimated the dissimilarity between the haplotype structures of two groups in R 2.15 applying the Morisita-Horn index [38]. Note that the two divergence indices D_TN_ and H_MH_ were obtained by estimating the average pairwise distances between the population of concern and all other populations in the dataset.

Additional candidate ecological and topographical parameters of *O. hupensis* to be used for the modeling of schistosomiasis risks were selected based on their potential relevance for the distribution and/or susceptibility of snail populations [16], [39]–[41] (see Table 1).

**Table 1:** Candidate topographical and ecological characters of *Oncomelania h. hupensis* used for the SESR modeling.

Parameter	Source/data transformation	Original resolution	Relevance
Elevation	SRTM3 90 m Digital Elevation Model	90 m	Main snail distribution parameter
Slope	SRTM3 90 m Digital Elevation Model	90 m	Main snail distribution parameter
Bioclimatic variable bio6 (minimum temperature of coldest month)	Global Climate database at www.worldclim.org [82]	1000 m	Lethal temperature for *O. hupensis* is −2.7°C [71]
Bioclimatic variable bio11 (mean temperature of coldest quarter)	Global Climate database at www.worldclim.org [82]	1000 m	The development of both snails and parasite larvae requires a minimum temperature [72], [83], [84]
Bioclimatic variable bio12 (annual precipitation)	Global Climate database at www.worldclim.org [82]	1000 m	Proxy for suitable snail habitat [84]
Bioclimatic variable bio16 (precipitation of wettest quarter)	Global Climate database at www.worldclim.org [82]	1000 m	Proxy for flooding, transporting and/or potentially drowning of snails
Euclidean distances to water bodies	Calculated in ArcMap 9.3 based on water body data in www.diva-gis.org	90 m	Proxy for suitable snail habitat and/or flooding
Normalized Difference Vegetation Index (NDVI)	Moderate Resolution Imaging Spectroradiometer (MODIS) data for 2000–2010, United States Geological Survey. Clouds were masked and the ten year average was calculated by using the raster 2.0–12 package [85] for R 2.15	250 m	Proxy for soil moisture [8] and therefore suitable snail habitat (wetlands)

All original resolutions were re-sampled to 500 m.

#### Model building

In order to identify the candidate characters that discriminate between endemic/non-endemic areas and to build and visualize our SESR model, we here used maximum entropy modeling as implemented in MaxEnt 3.3.3k [42]. The software has been shown to perform well in species distribution [43] and disease modeling analyses [44]. The MaxEnt algorithm fits predictor variables (i.e., our snail traits) to the endemic state by discriminating between the 31 populations located within previously defined endemic areas [5] and 10,000 random extralimital points. Our genetic data were based on 45 distinct collection points and hence did not have the continuity of the topographical and ecological characters. We therefore first interpolated the gaps between populations using Inverse Distance Weighting (IDW) [45] as implemented in the gstat 1.0–14 package [46] for the R statistical environment. The IDW algorithm estimated values for the population indices by weighting the information of the nearest twelve sampling points with distance to the respective grid cell.

As correlated variables may lead to a decrease of model quality [47], we first tested similar variables (the four genetic variables, bio06 vs. bio11, and bio12 vs. bio16) in R for potential correlation using a conservative Pearson's r of 0.8 *sensu* Elith et al. and Rodda et al. [48], [49] as threshold. Whereas no correlation could be detected among the genetic variables, variable pairs bio6/bio11 and bio12/bio16 were correlated with r = 0.89 and r = 0.85, respectively. The final selection of the best combination of topographical, ecological and genetic candidate variables was done in MaxEnt by determining the area under the receiver operator curve (AUC), with increasing numbers of variables being penalized. In order to account for the presence of two pairs of correlated variables, we ran four individual MaxEnt analyses (all uncorrelated variables+bio06+bio12; all uncorrelated variables+bio06+bio16; all uncorrelated variables+bio11+bio12; and all uncorrelated variables+bio11+bio16). For each variable combination, 5-fold cross validation was done with 50 repeats. Individual variable contributions were evaluated by jacknife testing of significant differences in AUC values, applying parametric bootstrapping with 10,000 replicates. The best combination of variables that fulfilled the quality criteria was then used for the final run of 500 predictions. In order to avoid false positive predictions [50], we assessed the risk threshold for each prediction using a receiver operator curve plot (ROC) [51] with predictions below the threshold being omitted. After calculating the mean of the 500 final run predictions, the information was processed with the GIS-package dismo 0.7–23 [52] in R to visualize our SESR model.

### Linking Model Data for Schistosomiasis Risk with Infection Data

To evaluate our model and to assess its epidemiological value, we compared model predictions to observed human infection rates at county levels. Total numbers of human cases for each county were derived from Zhang et al. [53] and are based on epidemiological studies conducted in 1999–2001, 2007 and 2008. Average human infection rates were then expressed as average ratio of total number of incidences and population sizes for the respective area and year. Year-specific human population sizes were obtained from the NASA Socioeconomic Data and Applications Center (SEDAC) available at http://sedac.ciesin.columbia.edu/gpw.

We note that this validation routine is not completely independent: the state of endemic areas used as response variable is based on local environment and prevalence of infection [5], with the latter being derived from cattle and/or human [13]. However, as we only used non-human and non-cattle traits for our subsequent risk modeling, a validation of this model by actual infection data can be informative.

We tested the infection dataset for spatial autocorrelation utilizing Moran's *I* Test [54]. Semiparametric Eigenvector filtering [55], as implemented in the R-package spdep 0.5–51 [56], was performed to quantify the potential effect of spatial autocorrelation. Then a linear regression analysis was conducted in R by linking the county level infection rates to the mean model prediction values for the respective areas. The anova function in R was used to assess the contribution of the explanatory variables relative to spatial eigenvectors and to correct the values accordingly.

## Results

### Genetic Indices

Genetic indices for individual populations are provided in Supporting Table S2. Our dataset of 530 specimens consisted of 212 haplotypes. Values for haplotype diversity (H_D_) and haplotype divergence (H_MH_) ranged from 0.0 to 1.0 and 0.801 to 1.0, respectively. Nucleotide diversity (π) and Tajima-Nei-distance (D_TN_) varied from 0.0 to 0.016 and from 0.0 to 0.019, respectively. Spatially explicit heat maps of genetic values for indices that were later used for the SESR modeling (i.e., D_TN_ and H_D_) are given in Supporting Figure S1.

### Correlation Analysis of Previously Defined Endemic Areas and Snail Genetics

#### Analysis of molecular variance (Amova)

The *Φ_CT_* value for individuals collected during different sampling periods (i.e., 1980s, 1990s, and 2000s; Amova A) was −0.016 (*p* = 0.464). As negative values should be interpreted as zero [57] and as the high *p*-value indicates that our hypothesis (i.e., that the means of the groups are equal) is not rejected, a possible bias in our SESR modeling caused by different sampling periods appears to be unlikely.

The variation among groups of populations collected in endemic vs. non-endemic areas (Amova B) explained 18.1% of the total variation (*Φ_CT_* = 0.18, *p*<0.001), the variation among populations within groups explained 40.4% (*Φ_SC_* = 0.49, *p*<0.001), and the variation within populations 41.6% (*Φ_ST_* = 0.58, *p*<0.001). As the *Φ_CT_* value is relatively high and as there is a significant partitioning of variance of snail parameters from populations belonging to endemic areas vs. extralimital populations, genetic characters appear to explain in part the endemic area state (see Goal 1).

#### Multivariate Regression Trees (MRT)

Overall, the spatially-explicit MRT explained 62.79% of the total variance in the dataset. The pre-defined first split between endemic and non-endemic areas contributed a substantial 24.14% to this variance, confirming the results of the Amova B (see previous section).

### SESR Modeling

**Figure 2:**
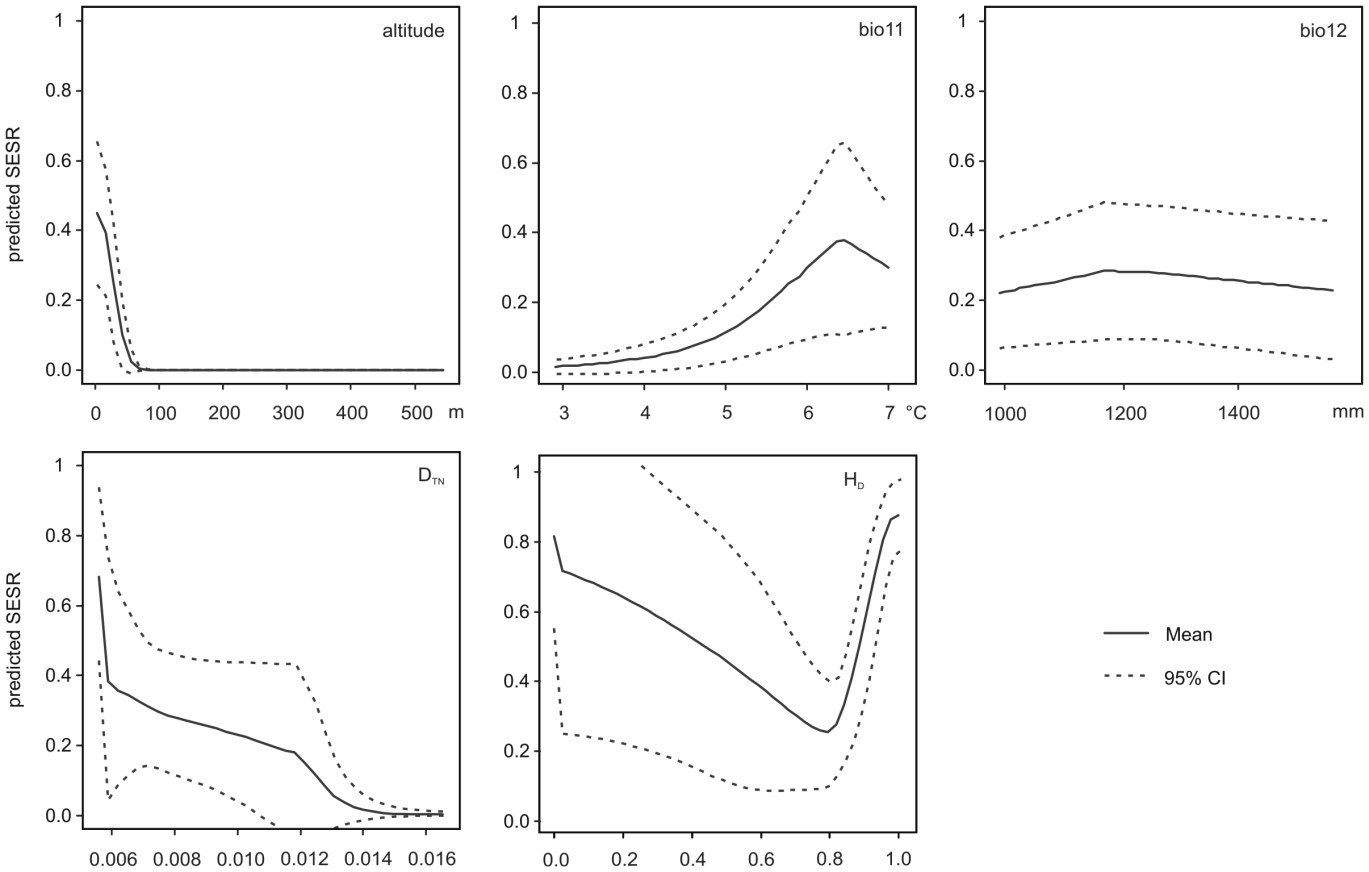
Individual response plots of five variables used for the SESR modeling. The plots were generated with the function response in the R-package dismo based on 500 model runs. Bio11 = mean temperature of coldest quarter, bio12 = annual precipitation, D_TN_ = Tajima-Nei-distance, H_D_ = haplotype diversity.

Based on the four individual MaxEnt runs and a possible combinations of a total of twelve candidate variables, the combination of altitude, bio11, bio12, D_TN_, and H_D_ was selected by MaxEnt (Goal 2; see Fig. 2).

**Figure 3:**
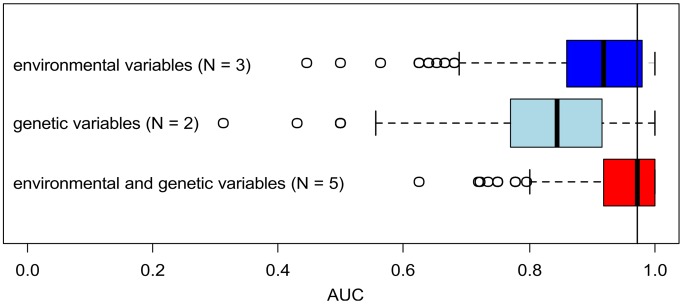
Results of jackknife testing of variable importance for the SESR modeling. The boxplots show the median goodness-of-fit values (AUC) of the models based on three environmental (bio11, bio12, altitude), two genetic (D_TN_, H_D_), and all five variables together with their respective 95% confidence limits (whiskers).

The goodness-of-fit (AUC) of this model had a median value of 0.97 (95% confidence limit: 0.80–1.00). When considering the two genetic and the three topographical/environmental parameters alone, the goodness-of-fit was 0.84 (0.57–1.00) and 0.92 (0.68–1.00), respectively (Fig. 3). The average goodness-of-fit value for two of the three topographical/environmental parameters was 0.86 (N = 3; details not shown here) and thus very similar to the value of the two genetic parameters (0.84). Parametric bootstrapping showed significant differences in means between the model with all variables and the model without genetic parameters. The mean risk threshold as indicated by the ROC plot was 0.115.

**Figure 4:**
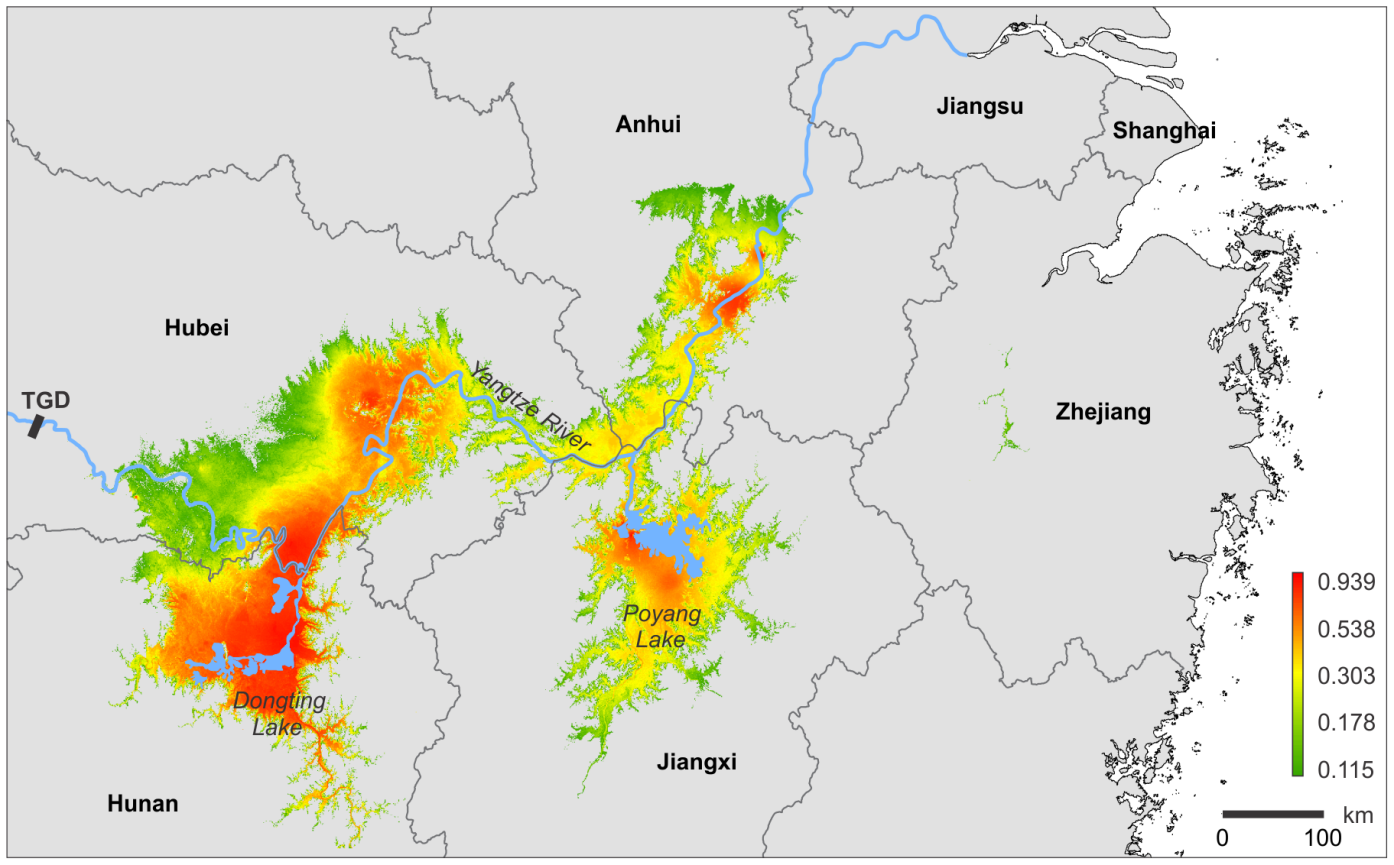
Output of the SESR modeling. Visualization of the schistosomiasis risk in eastern China (green color: low risk; red color: high risk). TGD = Three Gorges Dam.

The visualization of the SESR model (Goal 3), conducted with the R-package dismo, is shown in Fig. 4. Accordingly, the predicted schistosomiasis risk is highest in the regions of (i) Dongting Lake and (ii) Poyang Lake, as well as in the Yangtze River floodplains and islands in (iii) Hanyang and Jianli counties (Hubei Province), and in (iv) Tongling and Guichi counties (Anhui Province). Moderate to high risks were inferred for the remaining floodplains of the Yangtze River in Hubei and Anhui provinces.

### Linking SESR Model Data with Human Infection Data

Our spatial risk model was compared to human infection rates [53] (Goal 4). Significant spatial autocorrelation was observed within a distance of 100 km using Moran's *I* test. The Anova conducted showed that linear regression including spatial eigenvectors significantly explained more variation than without (r^2^ = 0.476 and 0.238, respectively). Removing spatial autocorrelation from the infection dataset resulted in a final adjusted r^2^ of 0.338 (*p*<0.001, N = 284). Thus, SESR model predictions are correlated with human infection rates.

## Discussion

The basic findings of our study were: (i) intermediate host genetic parameters inferred from the COI gene are correlated with the distribution of previously defined endemic areas (Goal 1); (ii) the maximum entropy modeling suggested five explanatory variables (altitude, bio11, bio12, D_TN_, and H_D_) to discriminate between endemic/non-endemic areas (Goal 2); (iii) the visualization of our SESR model indicated the highest risks for the regions of Dongting and Poyang lakes as well as some floodplains of the Yangtze River in Hubei and Anhui provinces (Goal 3); and (iv) our model predictions are correlated with human infection rates (Goal 4). These findings are discussed further below in relation to the spatial distribution of endemic areas, the quality of predictive intermediate host traits (particularly genetic traits), and the implications of our risk modeling for future schistosomiasis surveillance and control strategies.

### High Risk Areas

Model predictions suggested four relatively distinct, yet not fully isolated, areas as high risk regions. These areas, and the lack of complete isolation thereof, fit relatively well the spatial distribution of schistosomiasis previously suggested based on Bayesian random-effect modeling of reported schistosomiasis cases [53]. The areas include the two major lake systems Dongting and Poyang, which are at the center of schistosomiasis control in eastern China [11]. They have long been considered to be endemic areas [58] and a high number of human re-infections occur there [13], [59]. Both lake regions are heavily affected by the annual flooding of the Yangtze River and the associated rainy season (July–September) that causes a considerable increase of their surface areas [60], [61]. These floods may have countervailing effects on the transmission of schistosomiasis in the lakes. Whereas they may result in large-scale drowning of adult snails and are therefore used in some regions as snail control measure [62], flooding and heavy rains generally promote schistosomiasis transmission in the region: (i) floods and associated sediment input create and sustain suitable snail habitat, (ii) floods are a major source of introduction and re-introduction of snails, (iii) floods lead to the admixture of different parasite lineages to which snail populations may not be well adapted, and (iv) inundation following heavy rains helps sustain suitable habitat for free-swimming parasite larvae.

Moreover, the lakes serve as major sediment traps for upstream Yangtze River sections during the flood season and as suppliers of suspended sediments for downstream river sections during the dry season [63]–[66]. In addition, the extensive floodplain areas of the lakes play an important role in flood control of downstream river sections. As a result, the parts of the Yangtze River upstream to the lakes are, generally, less suited as snail habitat than downstream parts. Our modeling confirmed these differential effects of the lakes on the Yangtze River. Whereas parts of the river upstream of the lakes only had low to medium risks, two high risk areas were located downstream of Lake Dongting (Hanyang and Jianli counties) and Lake Poyang (Tongling and Guichi counties). Overall, the enhancing effect of Lake Dongting seemed to be larger than that of Lake Poyang (Fig. 4). This could be explained with the upstream position of Lake Dongting. However, this could also partly result from a slight sampling bias (i.e., we studied comparable few snail populations from Yangtze River sections downstream of Lake Poyang). Interestingly, the high risk regions inferred are not completely isolated. Therefore, re-introductions may play an important role for maintaining infections in populations [7] (also see section below).

In summary, the SESR modeling indicated four interconnected areas in the lower Yangtze River basin with high probabilities of disease occurrence. Risk values can vary considerably on small scales (i.e., within few kilometers) and are thus not associated with administrative entities. We suggest the complex hydrological interplay of lakes Dongting and Poyang with upstream and downstream sections of the Yangtze River in space and time as an important driver for the maintenance of the disease in eastern China.

### Predictive Variables for SESR

Several studies have identified variables associated with schistosomiasis risk based on the life cycle of *S. japonicum* [2], [39], [40], [67]–[70]. However, none of these studies utilized genetic characters, although their predictive value has long been suggested (see below). Our results confirmed the value of intermediate host genetic information for risk modeling. Two of the five final risk model variables were genetic characters (Fig. 2). In fact, the parameter with the single highest predictive value was haplotype diversity (H_D_). In addition to this diversity parameter, the model also suggested a divergence index (D_TN_) as risk variable, confirming the assumption that for understanding endemic areas and the potential isolation thereof, both genetic diversity (i.e., differences within populations) and divergence parameter (i.e., differences between populations) are of interest. In fact, one of the key findings of this study is that low divergence values indicate high risks (Fig. 2). In other words, not spatial isolation of endemic populations but high levels of gene flow and/or local effective population sizes drive schistosomiasis. This has been hypothesized before within the framework of frequency dependent selection and/or in the context of demographic effects. The annual flooding of the Yangtze River, for example, may not only cause high levels of gene flow in snail but also in parasite populations, potentially leading to multiple infections or infection with parasites to which the snails are not adopted locally [16], [19], [24].

The remaining three variables—altitude, mean temperature of coldest quarter (bio11), and annual precipitation (bio12)—are all environmental (note that bio11 and bio12 are correlated with bio06 and bio16, respectively). These and several related variables have been previously suggested to be risk indicative [68], [69], [71]. In contrast, other environmental variables previously found to be important, such as water availability and vegetation index [68], [72], did not significantly improve the goodness-of-fit of our model.

Overall, the relative contribution of the genetic parameters is very similar to the predictive power of the environmental ones. However, given the already high values of environmental and genetic parameters alone, complementing genetic with ecological variables and vice versa resulted in a significant but only slight increase in total goodness-of-fit. Nonetheless, genetic parameters appear to be particularly important as predictive variables as their application considerably reduces the confidence intervals of the predictions compared to environmental variables alone (i.e., by >40%; Fig. 3). As their addition helps improving the accuracy of the model, they are particularly helpful in areas with a high spatial risk dynamic. There, genetic parameters will likely enhance local risk modeling.

In summary, our results indicate that genetic intermediate host parameters can explain the endemic area state as well as the underlying evolutionary processes (i.e., ‘population’ isolation vs. admixture). The results also demonstrate the value of these parameters for risk modeling and for improving the local accuracy of model predictions. Both divergence and diversity parameters are of interest, and genetic and environmental parameters can be complementary in such an analysis.

### Implications of SESR Modeling for Schistosomiasis Surveillance and Control Strategies

Our findings have several implications for schistosomiasis surveillance and control in China, particularly under the current situation of decreasing overall disease transmission rates and increasing potential for re-emergence:

- With quantitative parasite data being extremely difficult to obtain, data of the co-evolved intermediate host can be used for risk modeling as previously suggested [16], [18].
- Given that risk values can vary over small distances, fine-scale units should be used in place of administrative boundaries when making surveillance and control decisions.
- High-risk areas as derived from risk models should receive surveillance and control priority, yet given the complex hydrological interplay of water bodies in the Yangtze River floodplains, information from extralimital areas should be considered as well for local strategies.
- As population genetic parameters of the intermediate host significantly increase the quality of risk predictions, future routine genetic surveys of snail populations are encouraged, particularly within and near high risk areas to better understand both local population structures as well as regional patterns of population isolation and exchange among lakes and rivers.

In summary, this study stressed the role of intermediate host traits for understanding schistosomiasis occurrence in China in a spatially-explicit manner. It also showed that these traits may serve as sensible proxies for infection risks and highlighted the potential of genetic characters for future risk modeling.

### Limitations and Outlook

This study extended the traditional ecological niche-modeling approach, which is frequently used to predict the occurrence of parasite and/or host species, to an approach for predicting the probability of disease occurrence. This was possible by two key modifications/additions. First, we included epidemiological data (i.e., the spatial distribution of endemic areas) that were used to discriminate against. In a recent study on the West Nile virus mosquito vector [73], the authors demonstrated that such an approach was useful for predicting human incidences of West Nile virus. They also suggested that this method for creating probability distribution maps could be applied to the study of other vector-borne diseases. Second, we accounted for the problem that traditional niche-modeling approaches are typically based on (extrinsic) environmental data and not on intrinsic evolutionary information of the actual target populations. We here attempted to overcome this problem by including evolutionary and demographically relevant genetic information of the intermediate host in our disease occurrence modeling. To our best knowledge, this is the first study on vector-borne diseases that used this approach.

Whereas our findings could have important consequences for future schistosomiasis surveillance and control strategies, such study with pilot character also has some limitations. Given the considerable genetic diversity of *O. hupensis* in mainland China [24], [74]–[76], our study of only 500+ specimens did likely not cover the full genetic structure of the intermediate snail host. Moreover, the mitochondrial COI gene used in this study can only reflect the phylogeographical and demographic history of populations; it is very likely not directly involved in co-evolutionary processes.

Therefore, we encourage future deep-sequencing based genomic and transcriptomic studies of the intermediate host that aim at identifying the genes responsible for susceptibility and/or resistance to infections (incl. horizontal gene transfer from parasite to snail). Furthermore, the view point of the intermediate host system as major transmission bottleneck has proven to be useful for understanding disease distribution. However, more specific evolutionary analyses such as identifying loci under selection (‘selective sweeps’) in high risk snail populations would help to better delineate co-evolutionary processes leading to rapid adaptation [77]. Using this information in future risk models will very likely further improve the predictive power of genetic information and may open new control perspectives. Finally, for an explicit modeling of actual infection risks in a multi-host parasite system, quantitative data on interspecies transmission dynamics are necessary [78], [79]. This, in turn, would require future cross-disciplinary studies focusing on host-parasite ecological networks that consider all participating species [79].

## Supporting Information

Figure S1 **Spatially explicit heat maps of genetic indices used for the SESR modeling.** Left: Tajima-Nei-distance (D_TN_); right: haplotype diversity (H_D_).
(TIF)

Table S1 **Locality information, number of specimens per site, and GenBank accession numbers for the total of 530 specimens of** ***Oncomelania h. hupensis*** **studied.** Populations located inside of endemic areas are marked with an asterisk.
(DOCX)

Table S2 **Genetic population indices for 45** ***Oncomelania h. hupensis populations*** **studied.**
(DOCX)
