# Supplementary figures and images for: Spatially Explicit Modeling of Schistosomiasis Risk in Eastern China Based on a Synthesis of Epidemiological, Environmental and Intermediate Host Genetic Data
Source: PLoS Negl Trop Dis. 2013 Jul 25;7(7):e2327. doi: 10.1371/journal.pntd.0002327 (PMC3723594; doi:10.1371/journal.pntd.0002327)

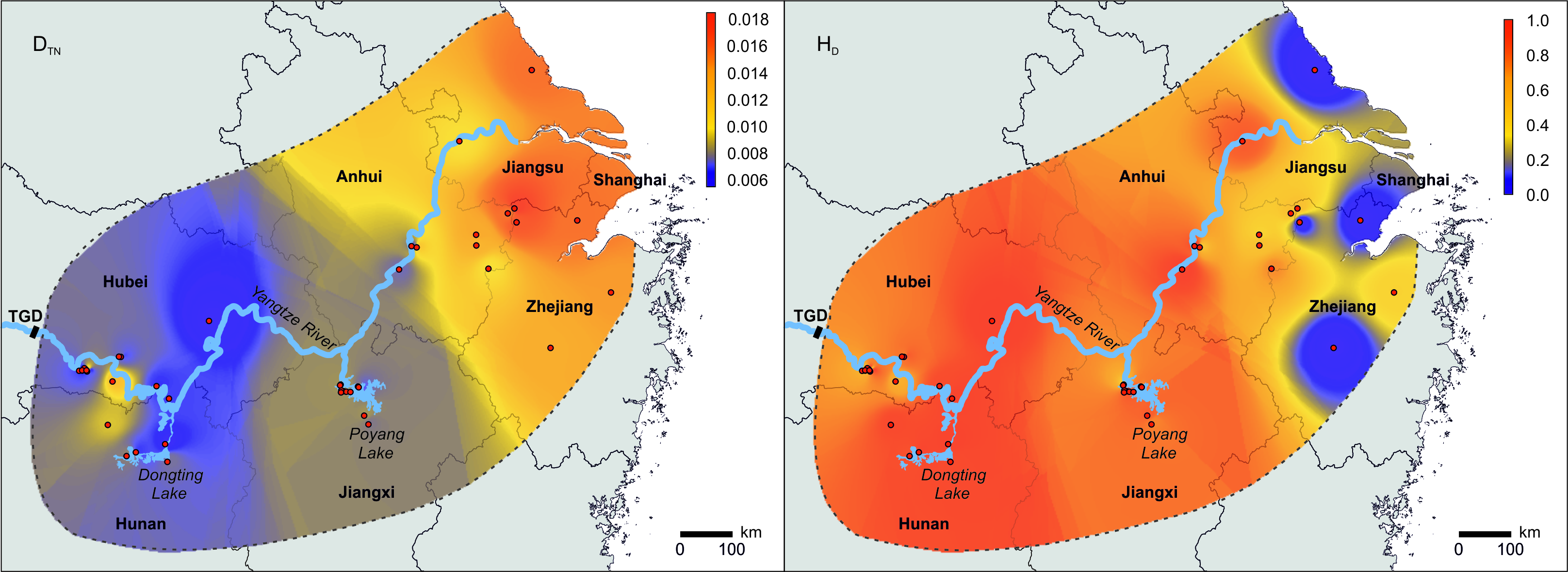

Supplement: Figure S1 — Spatially explicit heat maps of genetic indices used for the SESR modeling. Left: Tajima-Nei-distance (DTN); right: haplotype diversity (HD). (TIF) [file pntd.0002327.s001.tif]
