# Supplementary material for: Spatially Explicit Modeling of Schistosomiasis Risk in Eastern China Based on a Synthesis of Epidemiological, Environmental and Intermediate Host Genetic Data
Source: PLoS Negl Trop Dis. 2013 Jul 25;7(7):e2327. doi: 10.1371/journal.pntd.0002327 (PMC3723594; doi:10.1371/journal.pntd.0002327)
Supplement: Table S1 — Locality information, number of specimens per site, and GenBank accession numbers for the total of 530 specimens of Oncomelania h. hupensis studied. Populations located inside of endemic areas are marked with an asterisk. (DOCX) [file pntd.0002327.s002.docx]

## Supporting Table S1. Locality information, number of specimens per site, and GenBank accession numbers for the total of 530 specimens of *Oncomelania h. hupensis* studied. Populations located inside of endemic areas are marked with an asterisk.

| Province/county | Locality code | Collection year | Latitude (°N) | Longitude (°E) | Number of specimens | GenBank accession # |
| --- | --- | --- | --- | --- | --- | --- |
| Anhui |  |  |  |  |  |  |
| Tongling | AN_I* | 2004/05 | 30.9495 | 117.7660 | 28 | KC989107–KC989134 |
| Guichi | AN_II* | 1997 | 30.6735 | 117.4549 | 10 | AF254500–AF254509 [81] |
| Tongling | AN_III* | 1997 | 30.9150 | 117.8509 | 11 | KC989135–KC989145 |
| Guangde | AN_IV | 1999 | 31.0490 | 119.5499 | 10 | AF254516–AF254525 [81] |
| Ninggou | AN_V | 1984 | 30.3730 | 118.9725 | 10 | AF254490–AF254499 [81] |
| Ninggou | AN_VI | 1998 | 30.7313 | 118.8716 | 4 | KC989146–KC989149 |
| Xuanzhou | AN_VII | 1997 | 30.8780 | 118.9143 | 5 | AF254546–AF254550 [81] |
| Hubei |  |  |  |  |  |  |
| Jianli | HB_I* | 1984 | 29.82 | 112.90 | 10 | KC989150–KC989159 |
| Songzi | HB_II* | 1998 | 30.205 | 111.765 | 10 | AF306572–AF306581 [20] |
| Songzi | HB_III | 1998 | 30.254 | 111.726 | 6 | AF306582–AF306587 [20] |
| Songzi | HB_IV | 1998 | 30.229 | 111.683 | 10 | AF306588–AF306597 [20] |
| Songzi | HB_V | 1998 | 30.233 | 111.684 | 5 | AF306598–AF306602 [20] |
| Songzi | HB_VI | 1998 | 30.232 | 111.631 | 10 | AF306603–AF306612 [20] |
| Songzi | HB_VII* | 1998 | 30.221 | 111.755 | 9 | AF306613–AF306621 [20] |
| Songzi | HB_VIII* | 1998 | 30.239 | 111.746 | 9 | AF306622–AF306630 [20] |
| Gong‘an | HB_IX* | 1996 | 30.0 | 112.2 | 5 | KC989160–KC989164 |
| Jianli | HB_X* | 1996 | 29.6167 | 113.0667 | 17 | AF254484–AF254489 [81], KC989165–KC989175 |
| Shashi | HB_XI* | 2004 | 30.3258 | 112.3544 | 18 | KC989176–KC989193 |
| Shashi | HB_XII* | 2004/05 | 30.3189 | 112.3886 | 42 | KC989194–KC989235 |
| Hanyang | HB_XIII* | 1984 | 30.5689 | 114.0169 | 10 | AF253072–AF253073, AF254478–AF254479 [81] |
| Hunan |  |  |  |  |  |  |
| Yuanjiang | HN_I* | 1996 | 28.97 | 112.34 | 16 | KC989242–KC989257 |
| Yuanjiang | HN_II* | 1996 | 29.0 | 112.9 | 19 | KC989258–KC989276 |
| Xiangyin | HN_III* | 1996 | 28.75 | 112.83 | 25 | KC989277–KC989301 |
| Jinshi | HN_IV* | 1996 | 29.42 | 111.94 | 8 | KC989302– KC989309 |
| Hanshou | HN_V* | 2004/05 | 28.9395 | 112.1668 | 39 | KC989310–KC989348 |
| Jiangsu |  |  |  |  |  |  |
| Nanjing | JG_I* | 1998 | 32.20 | 119.04 | 16 | KC989349–KC989364 |
| Dongtai | JG_II | 1999 | 32.891 | 120.646 | 9 | AF254526–AF254531 [81], KC989365–KC989367 |
| Jiangxi |  |  |  |  |  |  |
| Jongxiu | JX_I* | 2004/05 | 29.1964 | 116.0545 | 40 | KC989368–KC989407 |
| Duchang | JX_II* | 1999 | 29.218 | 116.289 | 8 | KC989408–KC989415 |
| Duchang | JX_III* | 1999 | 29.208 | 116.298 | 4 | KC989416–KC989419 |
| Jongxiu | JX_IV* | 1999 | 29.293 | 115.994 | 3 | KC989420–KC989422 |
| Xingzi | JX_V* | 1999 | 29.299 | 116.006 | 13 | KC989426–KC989438 |
| Jongxiu | JX_VI* | 1999 | 29.209 | 116.04 | 7 | KC989439–KC989445 |
| Jongxiu | JX_VII* | 1999 | 29.204 | 116.035 | 3 | KC989446–KC989448 |
| Jongxiu | JX_VIII* | 1999 | 29.199 | 116.033 | 6 | KC989449–KC989454 |
| Xingzi | JX_IX* | 1999 | 29.196 | 116.055 | 3 | KC989423–KC989425 |
| Jongxiu | JX_X* | 1999 | 29.198 | 116.051 | 8 | KC989455–KC989462 |
| Jongxiu | JX_XI* | 1999 | 29.199 | 116.041 | 2 | KC989463–KC989464 |
| Jinxian | JX_XII* | 1999 | 28.682 | 116.32 | 10 | KC989465–KC989474 |
| Yugan | JX_XIII* | 1997 | 28.802 | 116.275 | 16 | KC989475–KC989490 |
| Zhejiang |  |  |  |  |  |  |
| Changxing | ZJ_I | 1999 | 30.8955 | 119.6562 | 9 | AF254532–AF254535 [81], KC989491–KC989495 |
| Changxing | ZJ_II | 1999 | 31.0931 | 119.685 | 9 | AF254510–AF254515 [81], KC989496–KC989498 |
| Jinhua Shì | ZJ_III | 1999 | 29.078 | 119.647 | 10 | KC989499–KC989508 |
| Shengsi | ZJ_IV | 1999 | 29.5927 | 120.9067 | 5 | KC989509–KC989513 |
| Xiuzhou | ZJ_V | 1999 | 30.6939 | 120.6894 | 3 | KC989514–KC989516 |
