# Supplementary material for: Spatially Explicit Modeling of Schistosomiasis Risk in Eastern China Based on a Synthesis of Epidemiological, Environmental and Intermediate Host Genetic Data
Source: PLoS Negl Trop Dis. 2013 Jul 25;7(7):e2327. doi: 10.1371/journal.pntd.0002327 (PMC3723594; doi:10.1371/journal.pntd.0002327)
Supplement: Table S2 — Genetic population indices for 45 Oncomelania h. hupensis populations studied. (DOCX) [file pntd.0002327.s003.docx]

## Supporting Table S2. Genetic population indices for 45 *Oncomelania h. hupensis populations* studied.

| Locality | Nucleotide diversity (π) | Tajima-Nei-distance (D_TN_) | Haplotype diversity  (H_D_) | Haplotype divergence  (H_MH_) |
| --- | --- | --- | --- | --- |
| AN_I | 0.016 | 0.008 | 0.960 | 0.964 |
| AN_II | 0.016 | 0.008 | 0.978 | 1.000 |
| AN_III | 0.001 | 0.012 | 0.467 | 1.000 |
| AN_IV | 0.003 | 0.016 | 0.511 | 1.000 |
| AN_V | 0.008 | 0.010 | 0.711 | 0.986 |
| AN_VI | 0.002 | 0.013 | 0.500 | 0.986 |
| AN_VII | 0.009 | 0.014 | 0.400 | 1.000 |
| HB_I | 0.009 | 0.007 | 0.911 | 0.951 |
| HB_II | 0.015 | 0.006 | 0.978 | 0.961 |
| HB_III | 0.011 | 0.011 | 0.933 | 1.000 |
| HB_IV | 0.013 | 0.006 | 0.644 | 0.959 |
| HB_IX | 0.008 | 0.013 | 0.600 | 1.000 |
| HB_V | 0.014 | 0.006 | 0.700 | 0.958 |
| HB_VI | 0.008 | 0.007 | 0.356 | 0.959 |
| HB_VII | 0.016 | 0.008 | 0.917 | 0.971 |
| HB_VIII | 0.008 | 0.011 | 0.417 | 0.972 |
| HB_X | 0.010 | 0.007 | 0.971 | 0.968 |
| HB_XI | 0.012 | 0.008 | 0.725 | 0.978 |
| HB_XII | 0.010 | 0.007 | 0.858 | 0.946 |
| HB_XIII | 0.011 | 0.006 | 1.000 | 0.974 |
| HN_I | 0.010 | 0.007 | 0.958 | 0.989 |
| HN_II | 0.011 | 0.006 | 0.977 | 0.978 |
| HN_III | 0.012 | 0.008 | 0.920 | 0.990 |
| HN_IV | 0.014 | 0.010 | 0.929 | 1.000 |
| HN_V | 0.008 | 0.008 | 0.881 | 0.980 |
| JG_I | 0.012 | 0.010 | 0.842 | 0.959 |
| JG_II | 0.000 | 0.016 | 0.000 | 0.979 |
| JX_I | 0.003 | 0.008 | 0.645 | 0.801 |
| JX_II | 0.002 | 0.009 | 0.679 | 0.811 |
| JX_III | 0.008 | 0.006 | 0.833 | 0.888 |
| JX_IV | 0.000 | 0.009 | 0.000 | 0.887 |
| JX_IX | 0.003 | 0.009 | 1.000 | 0.827 |
| JX_V | 0.001 | 0.009 | 0.641 | 0.801 |
| JX_VI | 0.003 | 0.008 | 0.952 | 0.870 |
| JX_VII | 0.002 | 0.008 | 1.000 | 0.879 |
| JX_VIII | 0.003 | 0.009 | 0.933 | 0.880 |
| JX_X | 0.002 | 0.009 | 0.607 | 0.828 |
| JX_XI | 0.002 | 0.009 | 1.000 | 0.823 |
| JX_XII | 0.003 | 0.008 | 0.733 | 0.822 |
| JX_XIII | 0.002 | 0.009 | 0.842 | 0.866 |
| ZJ_I | 0.000 | 0.017 | 0.000 | 1.000 |
| ZJ_II | 0.002 | 0.019 | 0.500 | 1.000 |
| ZJ_III | 0.000 | 0.014 | 0.000 | 1.000 |
| ZJ_IV | 0.001 | 0.015 | 0.400 | 0.935 |
| ZJ_V | 0.000 | 0.016 | 0.000 | 0.978 |
